# Trends in incidence and prevalence of type 1 diabetes between 1999 and 2019 based on the Childhood Diabetes Registry of Saxony, Germany

**DOI:** 10.1371/journal.pone.0262171

**Published:** 2021-12-31

**Authors:** Ulf Manuwald, Olaf Schoffer, Joachim Kugler, Henna Riemenschneider, Thomas Michael Kapellen, Wieland Kiess, Ulrike Rothe

**Affiliations:** 1 Health Sciences/Public Health, Faculty of Medicine “Carl Gustav Carus”, Technische Universität Dresden, Dresden, Germany; 2 Center of Evidence-Based Healthcare, University Hospital “Carl Gustav Carus”, Technische Universität Dresden, Dresden, Germany; 3 Department of General Practice/MK3, Faculty of Medicine “Carl Gustav Carus”, Technische Universität Dresden, Dresden, Germany; 4 Department of Women and Child Health, Center for Pediatric Research, Hospital for Children and Adolescents, University of Leipzig, Leipzig, Germany; 5 Median Childrens Hospital Bad Kösen, Bad Kösen, Germany; Chung Shan Medical University, TAIWAN

## Abstract

**Objective:**

The Childhood Diabetes Registry of Saxony has been existing since 1999. The aim of this study was to investigate the incidence rates, cohort and point prevalence, and the trends of type 1 diabetes among children and adolescents based on the registry data over the past 21 years.

**Methods:**

A completeness check of the Childhood Diabetes Registry of Saxony for the observation period 2012–2019 was performed using the capture-recapture method. The age-standardized incidence rates per 100,000 person years (PY) were estimated for the observation period 1999–2019. Prevalence was estimated per 100,000 children and adolescents as the point prevalence of five consecutive years, and as a cohort prevalence for the birth cohorts, which result from the difference of age and year at diagnosis. Trend analyses were executed using join point regression.

**Results:**

A completeness of 98% (95% CI 89–100) was determined for the period from 2012 to 2019. The standardized incidence rate of type 1 diabetes among children and adolescents increased from 17.1 per 100,000 PY in 1999 to 24.7 per 100,000 PY in 2019. If this trend continues, the incidence rate will increase to 34.8 (95% CI 24.4–49.6) per 100,000 PY in 2030. The point prevalence of 5 consecutive years did not show a continuous trend over time. According to this method, the prevalence reached a plateau in the last segment (2013–2019). The calculation of cohort prevalence indicated a continuous increase from 2013 to 2019 with no significant statistical difference in terms of sex.

**Conclusion:**

The point prevalence and the last incidence rates indicate that type 1 diabetes of children and adolescents is slowing down or has reached a plateau in Saxony. Nevertheless, the cohort prevalence predicts a steady increase. Future studies should continue investigating these trends in a longer observation period and consider including possible correlating environmental factors.

## Introduction

Type 1 diabetes is an autoimmune disease which etiology is widely unknown [1]. In addition to genetic predisposition and immunological factors, lifestyle and environmental factors are considered to have an effect on the development of type 1 diabetes [2–6].

Incidence of autoimmune diseases in general has been increasing worldwide over the last decades [7]. The incidence of type 1 diabetes has been increasing as well [8–10], but not constantly over time and regions [11–13]. Also in Europe, the incidence of type 1 diabetes is not homogenously distributed [3, 10, 12]. In some countries, the incidence and prevalence over time seems to have reached a plateau or stagnation or a reversal point [14–17]. One of the possible reasons for that could be environmental changes. For example, in the former Eastern Bloc countries, the adoption of the Western lifestyle occurred after the collapse of the political system in the late 20^th^ century. At that time, the increase of the (previously very low) incidence rates and prevalence of type 1 diabetes were observed more in Eastern than in Western Europe [18].

The Childhood Diabetes Registry of Saxony has collected data for 21 years, since 1999. In that time, the incidence of type 1 diabetes among children has increased threefold [19].

The population-based registry for children and adolescents with a high completeness of 97% for children 0 to 14 years in 2007/2008 is monitoring the development of type 1 diabetes incidence rates among children [20].

This new study aims to reassess the completeness of the registry more than 10 years after the previous study [20]. Further, the aim is to investigate, whether there has been a change regarding incidence trends after the previous evaluation by Manuwald et al. [18]. Furthermore, the data from 7 complete years allows a calculation of the cohort prevalence for the first time. In addition, the point prevalence will be calculated. Subsequently, based on the ascertained trends, a prediction of incidence and prevalence up to 2030 will be estimated.

## Methods

### Data source

In Saxony, all children with type 1 diabetes are referred to pediatric diabetologists working at the 31 pediatric hospitals that report the respective data to the Childhood Diabetes Registry of Saxony. For this study, the data of the Childhood Diabetes Registry of Saxony were analyzed for children and adolescents aged 0–14 years with type 1 diabetes from 1999 to 2019. Since the 1990s, type 1 diabetes has been defined according to the EURODIAB criteria [21].

### Comparison data source

A comparison (second) data was collected by resident physician practices with pediatric diabetes patients in the Dresden region for checking the completeness of the Childhood Diabetes Registry of Saxony (for more details, please see the S1 Table).

### Population at risk

Population data regarding children aged 0–14 were obtained from the Statistical State Office of Saxony for the years 1999 to 2016. The Free State of Saxony is a state located in the East of Germany with about 4,078 million inhabitants in 2019. In Saxony, the population under the age of 15 years decreased from 578,355 (100%) in 1999 to 436,305 (75%) in 2005. Since 2005, the population under the age of 15 years has slowly increased to 544,082 in 2019.

### Statistical methods

A new ***completeness check*** of the Childhood Diabetes Registry of Saxony for the observation period 2012–2019 was performed using the capture-recapture method (C-R method) [22]. The ***comparison data*** was collected in 2020 for patients with existing type 1 diabetes in 2018 and 2019 in practices located in a closed region of Saxony with postal codes from 010XX to 017XX.

***Incidence rates and prevalence*** were described based on direct age standardization procedures [23]. The incidence rates were estimated for each calendar year within the observations period 1999–2019. All incidence rates are age-standardized using the Standard New European Population (www.gbe-bund.de). Incidence data were presented as age-standardized incidence rates per 100,000 person-years (PY) with 95% confidence intervals [CI] estimated using the normal approximation.

Prevalence was estimated in two different ways: On the one hand, as the point prevalence of five consecutive years, and on the other hand, as cohort prevalence for the birth cohorts, which result from the difference of age and year at diagnosis.

These calculations were performed with the statistical software R (version 3.6.0).

***Trend analyses*** for incidence rates and prevalence were executed using join point regression, which is broadly used in cancer epidemiology [23]. Annual percent change (APC), average annual percent change (AAPC) and the respective 95% CI were estimated for the complete observations between 1999 and 2019. The fitted trend function is ln(y) = mx+b. Based on the slope parameter m the annual percent change (APC) is the transformation (exp(m)-1)*100. Potential trend changes over time were investigated for the complete time period. The AAPC is the average of APCs for distinct time periods with different trends. These calculations were performed with the Joinpoint Regression Program (version 4.2.0.2, Statistical Research and Applications Branch, National Cancer Institute, Bethesda, Maryland, USA).

***Forward projections*** for incidence rates and prevalence were derived using the fitted trend function. If there were trend changes over time, the trend from the last segment was projected forward.

### Ethics statements

The Childhood Diabetes Registry of Saxony was approved by the Ethical Committee of the Medical Faculty of the University of Leipzig (Reg. Nr. 236/21-ek), and a written informed consent was obtained from all parents or guardians of the involved children.

Only anonymous data were available for this analysis.

## Results

In the observation period of 21 years (1999–2019), a total of 2,155 cases (1,188 boys and 967 girls) younger than 15 years with onset of type 1 diabetes were recorded in the registry.

### Completeness of the Childhood Diabetes Registry of Saxony

In the comparison data, 50 patients with manifested type 1 diabetes between 2012 and 2019 were found. Only one patient was not listed in the Childhood Diabetes Registry of Saxony. Thus, we calculated a completeness of 98% (95% CI 89–100) for the period from 2012 to 2019 (S2 Table).

### Analysis of incidence and prevalence

**Fig 1:**
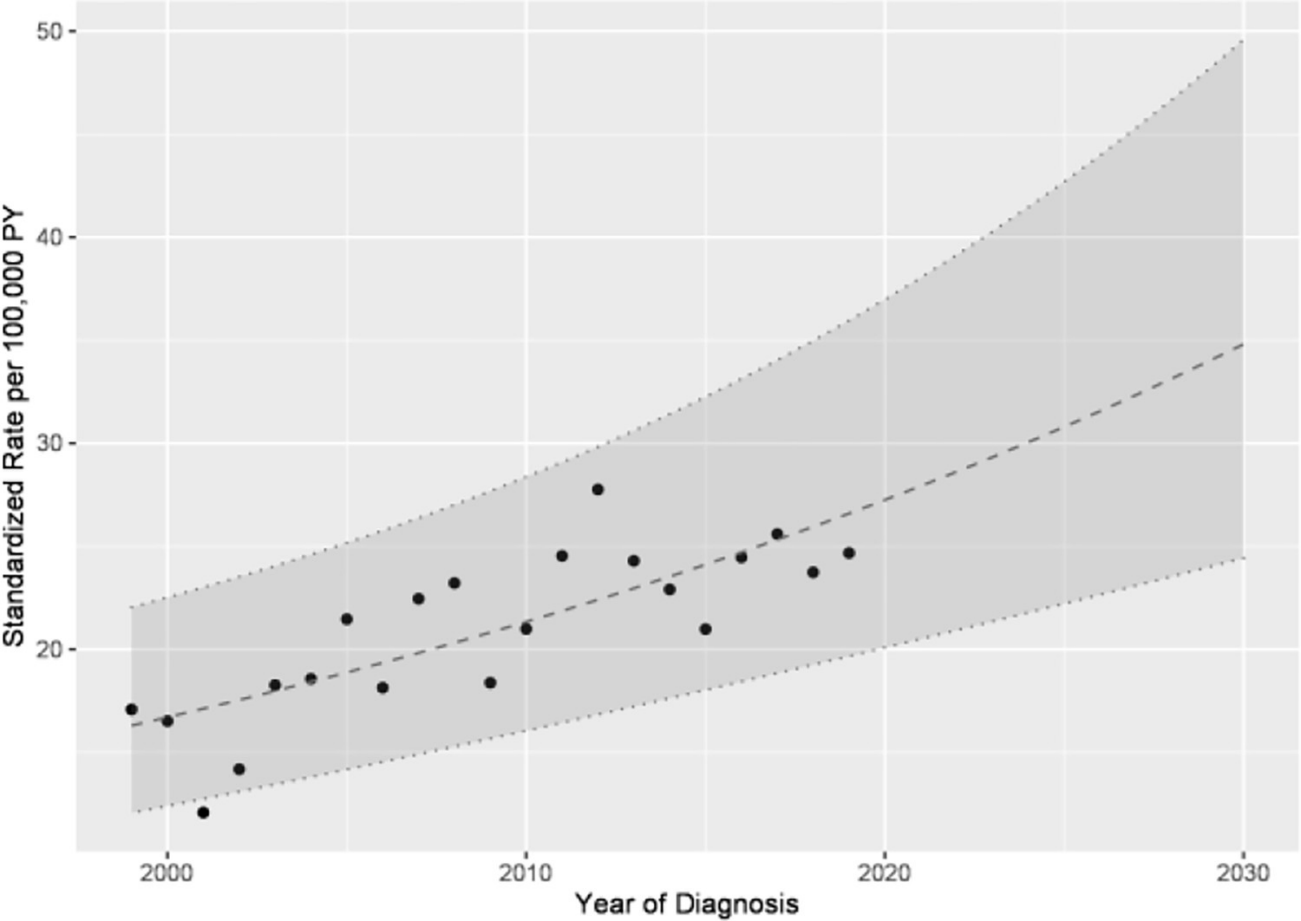
Incidence rates and forward projection of type 1 diabetes among children under 15 years of age in Saxony.

The analysis of ***incidence and prevalence*** revealed that the standardized incidence rate of type 1 diabetes increased from 17.1 per 100,000 PY in 1999 to 24.7 per 100,000 PY in 2019 (Fig 1). If this trend continues, the incidence rate of type 1 diabetes will increase to 34.8 (95% CI 24.4–49.6) per 100,000 PY in 2030 (S3 Table).

**Fig 2:**
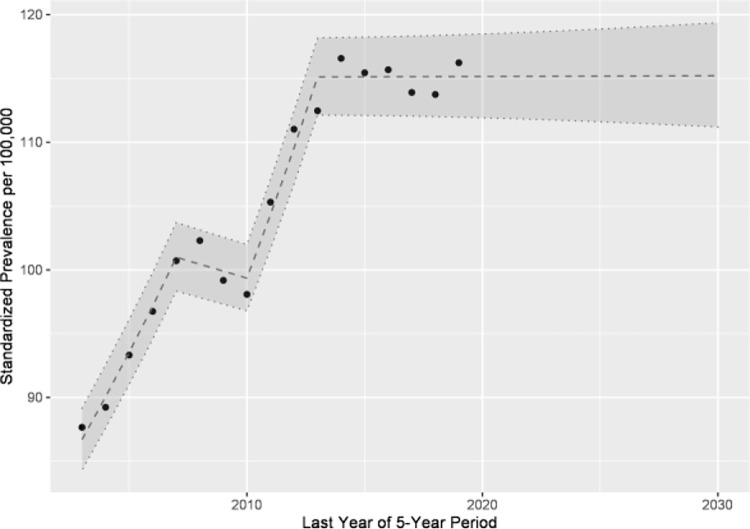
Point prevalence or 5-year prevalence and forward projection of type 1 diabetes among children under 15 years of age in Saxony.

The point prevalence of 5 consecutive years did not show a continuous trend over time (Fig 2). The join point regression yielded 3 join points. According to this method, the prevalence reached a plateau in the last segment 2013–2019 (Table 1). Considering the genders separately, no join point could be determined for the male children with type 1 diabetes and only one join point for the female children (Table 1).

**Table 1:** Results of the trend analysis for incidence and prevalence of type 1 diabetes with join point regression by gender and total. In the case of trend changes over time, the trends are given per time segment.

Measure (period)	Gender	Segment	APC	APC 95%-CI	AAPC	AAPC 95%-CI
Incidence (1999–2019)	female	complete period	2.3	0.9–3.7	2.3	0.9–3.7
	male		2.6	1.5–3.8	2.6	1.5–3.8
	total		2.5	1.5–3.4	2.5	1.5–3.4
Point prevalence (2003–2019)	female	2003–2015	2.1	1.3–2.9	1.1	0.1–2.1
	female	2015–2019	-2.0	-5.6–1.8		
	male	complete period	2.1	1.8–2.5	2.1	1.8–2.5
	total	2003–2007	3.9	2.5–5.2	1.8	0.9–2.6
	total	2007–2010	-0.5	-4.3–3.3		
	total	2010–2013	5.0	1.3–8.8		
	total	2013–2019	0.0	-0.6–0.6		
Cohort prevalence (2013–2019)	female	complete period	3.5	-4.0–11.5	3.5	-4.0–11.5
	male	complete period	3.7	-4.0–12.1	3.7	-4.0–12.1
	total	2013	3.6	-3.7–11.4	3.6	-3.7–11.4

(APC) annual percent change, (AAPC) average annual percent change, (CI) confidence interval

**Fig 3:**
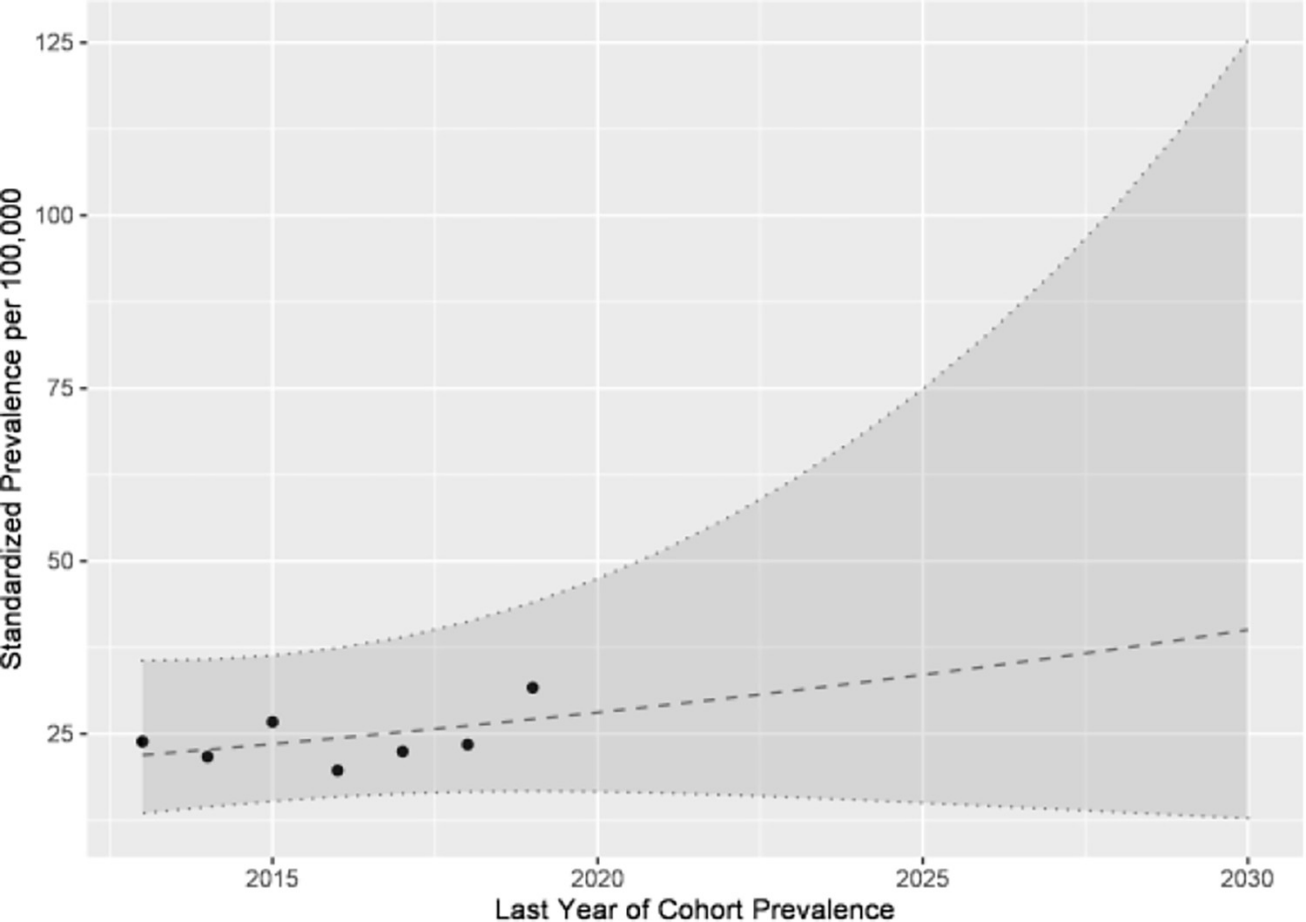
Cohort prevalence and forward projection of type 1 diabetes among children under 15 years of age in Saxony.

The calculation of cohort prevalence indicated a continuous increase from 2013 to 2019 with no significant statistical difference in terms of gender (Fig 3 and Table 1).

## Discussion

This study is based on data from the population-based Childhood Diabetes Registry of Saxony with a high data quality and completeness and with a long observation period of 21 years during which data were collected in the registry.

The above described change in environmental and lifestyle factors in the former Eastern Bloc countries probably led to an initial high increase in type 1 diabetes incidence rates that slowed over time [12].

Comparing the average annual percent change (AAPC) of 3.5 (95% CI 2.1–5.0) of the incidence of type 1 diabetes from Manuwald et al. [18] with the AAPC of 2.4 (95% CI 1.5–3.4) of the present evaluation (Table 1), a less steep increase over the whole observation period can be noticed descriptively, but the difference is not significant. This might result of that the previous estimation was made with fewer data points and was therefore more uncertain than the current one. Alternatively, it could be based on the last data points of the incidences of the current study, which could indicate a further flattening in the future. On the other side, the point prevalence after the last join point in 2013 have been resulting in a plateau.

In other countries, different trends of incidence rates of type 1 diabetes have been observed. Parviainen et al. [14] state that the incidence of type 1 diabetes among children aged 0–14 decreased between 2003 and 2018 in Finland [14]. In this context, the authors refer to a correlation between the development of type 1 diabetes and environmental factors such as adding vitamin D to dairy products [14]. In Sweden and the Czech Republic the incidence rates have reached a plateau of incidence [16, 17]. Based on point prevalence, the results of the current study also suggest a plateau (Fig 2).

Although genetic background is considered to be the primary risk factor for the development of type 1 diabetes, changes in incidence seem to be more affected by the influence of environmental factors [3]. According to Kondrashova et al. [24], the incidence of type 1 diabetes (1990 to 1999) in Russian Karelia among children and adolescents aged 0–14 years with Finnish ancestry was 11.1 (95% CI 0–24.8). In contrast, the incidence among children and adolescents in Finnish Karelia was 41.4 (95% CI 37.3–45.6). Since there are only minor genetic differences in these populations, Kondrashova et al. [24] suggest that socioeconomic influences, such as housing, hygiene, and diet, may play a major role in development of type 1 diabetes. Environmental influences may act as causative, accelerating, or protective factors in relation to type 1 diabetes [3]. Therefore, future studies should include these possible environmental factors in the evaluation to better uncover potential causes.

### Strengths and limitations

#### Strengths

The high completeness of the registry of 98% determined in this evaluation follows on from the previous completeness checks, which also determined a high completeness [20, 25]. Due to the high completeness of data of children and adolescents with type 1 diabetes in the Childhood Diabetes Registry of Saxony and the long observation period of 21 years, the informative power is correspondingly high.

Thus, not only longer incidence series but also–for the first time—trend observations for prevalence and thus first observations for several complete consecutive cohort prevalence are possible.

#### Limitations

Based on the 7 years of the complete cohort prevalence data and the large CIs of the projected cohort prevalence, only uncertain statements regarding the development in the future are possible. More certain statements about the future will be possible in a few years. The variance estimates should be treated with caution because of the rare occurrence of type 1 diabetes cases among children and the short observation period, but also since only data from the children aged from 0 to 14 years was available. The explanatory power of the variance estimates is limited based on the amount of incidence and prevalence data, which are affected by various factors. Unfortunately, there was no data available that could explain the triggering factors such as individual environmental factors or social parameters.

## Conclusions

The point prevalence and the last incidence rates indicate that type 1 diabetes of children and adolescents is slowing down or has reached a plateau in Saxony. Nevertheless, the cohort prevalence predicts a steady increase. However, future studies should continue investigating these trends within a longer observation period. In addition, register data should be completed with environmental and social data to explain the causes and trends of incidence and prevalence of type 1 diabetes of children and adolescents.

## Supporting information

S1 Table Details of the inclusion and exclusion criteria of the comparison data source. (DOCX)

S2 Table Completeness of the Childhood Diabetes Registry of Saxony with the capture-recapture method. (DOCX)

S3 Table Incidence rates and prevalence as well as prognosis of type 1 diabetes were estimated for each calendar year between the observations period 1999–2019. (DOCX)
