# Supplementary material for: Trends in incidence and prevalence of type 1 diabetes between 1999 and 2019 based on the Childhood Diabetes Registry of Saxony, Germany
Source: PLoS One. 2021 Dec 31;16(12):e0262171. doi: 10.1371/journal.pone.0262171 (PMC8719733; doi:10.1371/journal.pone.0262171)
Supplement: S1 Table — (DOCX) [file pone.0262171.s001.docx]

*Inclusion criteria for physician practices:*

1. pediatric practices

2. diabetology practices

3. general practitioners of internal medicine and general medicine

4. practices are located in a closed region in Saxony with postal codes from 010XX to 017XX

*Inclusion criteria for patients:*

1. with an existing type 1 diabetes (T1DM) in 2018 and 2019.

2. aged < 18 years

*Exclusion criteria for patients:*

1. cases without diagnosis of T1DM and other types of diabetes

2. patients without residence in Saxony
