# Supplementary material for: Trends in incidence and prevalence of type 1 diabetes between 1999 and 2019 based on the Childhood Diabetes Registry of Saxony, Germany
Source: PLoS One. 2021 Dec 31;16(12):e0262171. doi: 10.1371/journal.pone.0262171 (PMC8719733; doi:10.1371/journal.pone.0262171)
Supplement: S2 Table — (DOCX) [file pone.0262171.s002.docx]

|  | |  | |  | |  | | |  | | | |  |  |  |
| --- | --- | --- | --- | --- | --- | --- | --- | --- | --- | --- | --- | --- | --- | --- | --- |
| **First data source** | | | | | | | | | | | | |  |  |  |
|  | |  | | **registered** | | **not registered** | | | **total** | | | |  |  |  |
| **Second data source** | | **registered** | | 49 | | 1 | | | 50 | | | |  |  |  |
|  |  | **not registered** | | 207 | | 4.2 | | |  | | | |  |  |  |
|  | | **Total** | | 256 | |  | | | 261.2 | | | |  |  |  |
|  | |  | |  | |  | | |  | | | |  |  |  |
| **Completeness of first data source** | | | |  | |  | | | 0.98 | | | |  |  |  |
|  |  | |  | |  | |  |  | |  |  |  |  |  |  |
