# Supplementary material for: Trends in incidence and prevalence of type 1 diabetes between 1999 and 2019 based on the Childhood Diabetes Registry of Saxony, Germany
Source: PLoS One. 2021 Dec 31;16(12):e0262171. doi: 10.1371/journal.pone.0262171 (PMC8719733; doi:10.1371/journal.pone.0262171)
Supplement: S3 Table — (DOCX) [file pone.0262171.s003.docx]

| **measure** | **gender** | **year** | **Incidence rate/**  **prevalence** | **prediction (95%CI)** |
| --- | --- | --- | --- | --- |
| Incidence | female | 1999 | 13.8 | **15.3 (10.0; 23.4)** |
|  |  | 2000 | 15.5 | **15.7 (10.3; 23.8)** |
|  |  | 2001 | 11.1 | **16.0 (10.6; 24.3)** |
|  |  | 2002 | 14.0 | **16.4 (10.9; 24.8)** |
|  |  | 2003 | 19.9 | **16.8 (11.2; 25.3)** |
|  |  | 2004 | 15.5 | **17.2 (11.5; 25.8)** |
|  |  | 2005 | 22.6 | **17.6 (11.7; 26.3)** |
|  |  | 2006 | 14.8 | **18.0 (12.0; 26.9)** |
|  |  | 2007 | 20.3 | **18.4 (12.3; 27.5)** |
|  |  | 2008 | 23.3 | **18.8 (12.6; 28.1)** |
|  |  | 2009 | 15.6 | **19.3 (12.9; 28.8)** |
|  |  | 2010 | 17.3 | **19.7 (13.2; 29.4)** |
|  |  | 2011 | 23.0 | **20.2 (13.5; 30.1)** |
|  |  | 2012 | 28.7 | **20.7 (13.8; 30.9)** |
|  |  | 2013 | 21.2 | **21.1 (14.1; 31.6)** |
|  |  | 2014 | 21,9 | **21.6 (14.4; 32.5)** |
|  |  | 2015 | 22,3 | **22.1 (14.7; 33.3)** |
|  |  | 2016 | 17,5 | **22.6 (15.0; 34.2)** |
|  |  | 2017 | 19,3 | **23.2 (15.3; 35.1)** |
|  |  | 2018 | 22.3 | **23.7 (15.6; 36.1)** |
|  |  | 2019 | 24.6 | **24.3 (15.9; 37.1)** |
|  |  | 2020 | NA | **24.8 (16.2; 38.1)** |
|  |  | 2021 | NA | **25.4 (16.5; 39.2)** |
|  |  | 2022 | NA | **26.0 (16.8; 40.4)** |
|  |  | 2023 | NA | **26.6 (17.0; 41.5)** |
|  |  | 2024 | NA | **27.2 (17.3; 42.8)** |
|  |  | 2025 | NA | **27.9 (17.6; 44.1)** |
|  |  | 2026 | NA | **28.5 (17.9; 45.4)** |
|  |  | 2027 | NA | **29.2 (18.2; 46.8)** |
|  |  | 2028 | NA | **29.9 (18.5; 48.3)** |
|  |  | 2029 | NA | **30.5 (18.7; 49.8)** |
|  |  | 2030 | NA | **31.3 (19.0; 51.4)** |
|  | male | 1999 | 20.2 | **17.4 (12.2; 24.7)** |
|  |  | 2000 | 17.5 | **17.8 (12.6; 25.3)** |
|  |  | 2001 | 13.1 | **18.3 (12.9; 25.9)** |
|  |  | 2002 | 14.4 | **18.8 (13.3; 26.5)** |
|  |  | 2003 | 16.7 | **19.3 (13.7; 27.1)** |
|  |  | 2004 | 21.5 | **19.8 (14.1; 27.8)** |
|  |  | 2005 | 20.4 | **20.3 (14.5; 28.4)** |
|  |  | 2006 | 21.3 | **20.8 (14.9; 29.1)** |
|  |  | 2007 | 24.6 | **21.4 (15.3; 29.9)** |
|  |  | 2008 | 23.1 | **22.0 (15.7; 30.6)** |
|  |  | 2009 | 21.1 | **22.5 (16.2; 31.5)** |
|  |  | 2010 | 24.6 | **23.1 (16.6; 32.3)** |
|  |  | 2011 | 26.0 | **23.7 (17.0; 33.2)** |
|  |  | 2012 | 26.9 | **24.4 (17.4; 34.1)** |
|  |  | 2013 | 27.3 | **25.0 (17.9; 35.0)** |
|  |  | 2014 | 23.9 | **25.7 (18.3; 36.0)** |
|  |  | 2015 | 19.7 | **26.4 (18.8; 37.0)** |
|  |  | 2016 | 31.1 | **27.1 (19.2; 38.1)** |
|  |  | 2017 | 31.6 | **27.8 (19.6; 39.2)** |
|  |  | 2018 | 25.1 | **28.5 (20.1; 40.4)** |
|  |  | 2019 | 24.8 | **29.3 (20.5; 41.6)** |
|  |  | 2020 | NA | **30.0 (21.0; 42.9)** |
|  |  | 2021 | NA | **30.8 (21.5; 44.2)** |
|  |  | 2022 | NA | **31.6 (21.9; 45.6)** |
|  |  | 2023 | NA | **32.5 (22.4; 47.1)** |
|  |  | 2024 | NA | **33.3 (22.9; 48.6)** |
|  |  | 2025 | NA | **34.2 (23.3; 50.1)** |
|  |  | 2026 | NA | **35.1 (23.8; 51.8)** |
|  |  | 2027 | NA | **36.0 (24.3; 53.4)** |
|  |  | 2028 | NA | **37.0 (24.8; 55.2)** |
|  |  | 2029 | NA | **38.0 (25.3; 57.0)** |
|  |  | 2030 | NA | **39.0 (25.7; 59.0)** |
|  | total | 1999 | 17.1 | **16.3 (12.1; 22.0)** |
|  |  | 2000 | 16.5 | **16.7 (12.4; 22.5)** |
|  |  | 2001 | 12.1 | **17.1 (12.7; 23.0)** |
|  |  | 2002 | 14.2 | **17.5 (13.1; 23.5)** |
|  |  | 2003 | 18.3 | **18.0 (13.4; 24.0)** |
|  |  | 2004 | 18.6 | **18.4 (13.8; 24.6)** |
|  |  | 2005 | 21.5 | **18.9 (14.2; 25.2)** |
|  |  | 2006 | 18.1 | **19.3 (14.5; 25.8)** |
|  |  | 2007 | 22.5 | **19.8 (14.9; 26.4)** |
|  |  | 2008 | 23.2 | **20.3 (15.3; 27.0)** |
|  |  | 2009 | 18.4 | **20.8 (15.7; 27.7)** |
|  |  | 2010 | 21.0 | **21.3 (16.0; 28.4)** |
|  |  | 2011 | 24.5 | **21.9 (16.4; 29.1)** |
|  |  | 2012 | 27.8 | **22.4 (16.8; 29.8)** |
|  |  | 2013 | 24.3 | **23.0 (17.2; 30.6)** |
|  |  | 2014 | 22.9 | **23.5 (17.6; 31.4)** |
|  |  | 2015 | 21.0 | **24.1 (18.0; 32.3)** |
|  |  | 2016 | 24.5 | **24.7 (18.4; 33.1)** |
|  |  | 2017 | 25.6 | **25.3 (18.8; 34.0)** |
|  |  | 2018 | 23.7 | **25.9 (19.3; 35.0)** |
|  |  | 2019 | 24.7 | **26.6 (19.7; 36.0)** |
|  |  | 2020 | NA | **27.3 (20.1; 37.0)** |
|  |  | 2021 | NA | **27.9 (20.5; 38.0)** |
|  |  | 2022 | NA | **28.6 (20.9; 39.1)** |
|  |  | 2023 | NA | **29.3 (21.4; 40.3)** |
|  |  | 2024 | NA | **30.1 (21.8; 41.5)** |
|  |  | 2025 | NA | **30.8 (22.2; 42.7)** |
|  |  | 2026 | NA | **31.6 (22.7; 44.0)** |
|  |  | 2027 | NA | **32.3 (23.1; 45.3)** |
|  |  | 2028 | NA | **33.1 (23.5; 46.7)** |
|  |  | 2029 | NA | **34.0 (24.0; 48.1)** |
|  |  | 2030 | NA | **34.8 (24.4; 49.6)** |
| Point prevalence | female | 2003 | 83.4 | **84.0 (76.4; 92.5)** |
|  |  | 2004 | 86.4 | **85.8 (78.1; 94.3)** |
|  |  | 2005 | 90.5 | **87.6 (79.9; 96.2)** |
|  |  | 2006 | 91.5 | **89.5 (81.6; 98.1)** |
|  |  | 2007 | 93.3 | **91.4 (83.4; 100.1)** |
|  |  | 2008 | 94.8 | **93.3 (85.3; 102.1)** |
|  |  | 2009 | 92.4 | **95.3 (87.1; 104.2)** |
|  |  | 2010 | 87.1 | **97.3 (89.0; 106.4)** |
|  |  | 2011 | 95.8 | **99.3 (90.9; 108.6)** |
|  |  | 2012 | 104.6 | **101.4 (92.8; 110.9)** |
|  |  | 2013 | 102.8 | **103.6 (94.7; 113.3)** |
|  |  | 2014 | 108.5 | **105.8 (96.7; 115.7)** |
|  |  | 2015 | 112.4 | **108.0 (98.6; 118.2)** |
|  |  | 2016 | 107.1 | **105.8 (96.5; 116.0)** |
|  |  | 2017 | 98.2 | **103.7 (94.5; 113.8)** |
|  |  | 2018 | 99.8 | **101.7 (92.5; 111.7)** |
|  |  | 2019 | 103.3 | **99.7 (90.6; 109.7)** |
|  |  | 2020 | NA | **97.7 (88.6; 107.7)** |
|  |  | 2021 | NA | **95.8 (86.7; 105.7)** |
|  |  | 2022 | NA | **93.9 (84.9; 103.8)** |
|  |  | 2023 | NA | **92.0 (83.0; 102.0)** |
|  |  | 2024 | NA | **90.2 (81.2; 100.2)** |
|  |  | 2025 | NA | **88.4 (79.4; 98.4)** |
|  |  | 2026 | NA | **86.7 (77.6; 96.7)** |
|  |  | 2027 | NA | **84.9 (75.9; 95.1)** |
|  |  | 2028 | NA | **83.3 (74.2; 93.4)** |
|  |  | 2029 | NA | **81.6 (72.5; 91.8)** |
|  |  | 2030 | NA | **80.0 (70.9; 90.3)** |
|  | male | 2003 | 91.9 | **95.0 (88.1; 102.4)** |
|  |  | 2004 | 92.1 | **97.0 (90.1; 104.5)** |
|  |  | 2005 | 96.1 | **99.1 (92.1; 106.6)** |
|  |  | 2006 | 102.0 | **101.2 (94.1; 108.7)** |
|  |  | 2007 | 108.0 | **103.3 (96.2; 111.0)** |
|  |  | 2008 | 109.7 | **105.5 (98.3; 113.3)** |
|  |  | 2009 | 105.8 | **107.8 (100.4; 115.6)** |
|  |  | 2010 | 108.7 | **110.1 (102.6; 118.1)** |
|  |  | 2011 | 114.5 | **112.4 (104.8; 120.6)** |
|  |  | 2012 | 117.3 | **114.8 (107.0; 123.1)** |
|  |  | 2013 | 121.9 | **117.2 (109.2; 125.8)** |
|  |  | 2014 | 124.5 | **119.7 (111.5; 128.5)** |
|  |  | 2015 | 118.5 | **122.3 (113.8; 131.3)** |
|  |  | 2016 | 124.1 | **124.8 (116.2; 134.2)** |
|  |  | 2017 | 128.9 | **127.5 (118.5; 137.2)** |
|  |  | 2018 | 127.1 | **130.2 (120.9; 140.2)** |
|  |  | 2019 | 128.7 | **133.0 (123.3; 143.4)** |
|  |  | 2020 | NA | **135.8 (125.8; 146.6)** |
|  |  | 2021 | NA | **138.7 (128.3; 149.9)** |
|  |  | 2022 | NA | **141.6 (130.8; 153.3)** |
|  |  | 2023 | NA | **144.6 (133.4; 156.8)** |
|  |  | 2024 | NA | **147.7 (136.0; 160.4)** |
|  |  | 2025 | NA | **150.8 (138.6; 164.2)** |
|  |  | 2026 | NA | **154.1 (141.3; 168.0)** |
|  |  | 2027 | NA | **157.3 (144.0; 171.9)** |
|  |  | 2028 | NA | **160.7 (146.8; 175.9)** |
|  |  | 2029 | NA | **164.1 (149.5; 180.0)** |
|  |  | 2030 | NA | **167.6 (152.4; 184.3)** |
|  | total | 2003 | 87.7 | **86.7 (84.3; 89.2)** |
|  |  | 2004 | 89.2 | **90.1 (87.6; 92.6)** |
|  |  | 2005 | 93.3 | **93.5 (91.0; 96.1)** |
|  |  | 2006 | 96.8 | **97.1 (94.6; 99.8)** |
|  |  | 2007 | 100.7 | **101.0 (98.3; 103.7)** |
|  |  | 2008 | 102.3 | **100.4 (97.8; 103.1)** |
|  |  | 2009 | 99.2 | **99.9 (97.3; 102.6)** |
|  |  | 2010 | 98.1 | **99.4 (96.8; 102.0)** |
|  |  | 2011 | 105.3 | **104.3 (101.6; 107.1)** |
|  |  | 2012 | 111.0 | **109.5 (106.7; 112.5)** |
|  |  | 2013 | 112.5 | **115.1 (112.1; 118.2)** |
|  |  | 2014 | 116.6 | **115.1 (112.1; 118.2)** |
|  |  | 2015 | 115.5 | **115.1 (112.1; 118.2)** |
|  |  | 2016 | 115.7 | **115.1 (112.1; 118.3)** |
|  |  | 2017 | 113.9 | **115.1 (112.0; 118.3)** |
|  |  | 2018 | 113.7 | **115.1 (112.0; 118.4)** |
|  |  | 2019 | 116.2 | **115.2 (112.0; 118.4)** |
|  |  | 2020 | NA | **115.2 (111.9; 118.5)** |
|  |  | 2021 | NA | **115.2 (111.9; 118.6)** |
|  |  | 2022 | NA | **115.2 (111.8; 118.6)** |
|  |  | 2023 | NA | **115.2 (111.7; 118.7)** |
|  |  | 2024 | NA | **115.2 (111.7; 118.8)** |
|  |  | 2025 | NA | **115.2 (111.6; 118.9)** |
|  |  | 2026 | NA | **115.2 (111.5; 119)** |
|  |  | 2027 | NA | **115.2 (111.5; 119.1)** |
|  |  | 2028 | NA | **115.2 (111.4; 119.2)** |
|  |  | 2029 | NA | **115.2 (111.3; 119.3)** |
|  |  | 2030 | NA | **115.2 (111.2; 119.4)** |
| Cohort prevalence | female | 2013 | 22.1 | **20 (12.3; 32.6)** |
|  |  | 2014 | 18.9 | **20.7 (13.1; 32.7)** |
|  |  | 2015 | 24.3 | **21.4 (13.8; 33.2)** |
|  |  | 2016 | 20.0 | **22.1 (14.4; 34.1)** |
|  |  | 2017 | 18.9 | **22.9 (14.8; 35.5)** |
|  |  | 2018 | 21.0 | **23.7 (15.0; 37.5)** |
|  |  | 2019 | 28.9 | **24.5 (15.0; 40.0)** |
|  |  | 2020 | NA | **25.4 (14.9; 43.0)** |
|  |  | 2021 | NA | **26.2 (14.7; 46.7)** |
|  |  | 2022 | NA | **27.1 (14.5; 50.9)** |
|  |  | 2023 | NA | **28.1 (14.1; 55.8)** |
|  |  | 2024 | NA | **29.1 (13.8; 61.4)** |
|  |  | 2025 | NA | **30.1 (13.4; 67.6)** |
|  |  | 2026 | NA | **31.1 (12.9; 74.7)** |
|  |  | 2027 | NA | **32.2 (12.5; 82.7)** |
|  |  | 2028 | NA | **33.3 (12.1; 91.6)** |
|  |  | 2029 | NA | **34.4 (11.7; 101.6)** |
|  |  | 2030 | NA | **35.6 (11.3; 112.7)** |
|  | male | 2013 | 25.7 | **23.9 (13.8; 41.3)** |
|  |  | 2014 | 24.4 | **24.8 (14.8; 41.4)** |
|  |  | 2015 | 29.1 | **25.7 (15.7; 42.0)** |
|  |  | 2016 | 19.5 | **26.6 (16.4; 43.3)** |
|  |  | 2017 | 26.0 | **27.6 (16.9; 45.2)** |
|  |  | 2018 | 25.8 | **28.7 (17.1; 48.0)** |
|  |  | 2019 | 34.4 | **29.7 (17.2; 51.5)** |
|  |  | 2020 | NA | **30.8 (17.0; 55.9)** |
|  |  | 2021 | NA | **32.0 (16.7; 61.2)** |
|  |  | 2022 | NA | **33.2 (16.4; 67.3)** |
|  |  | 2023 | NA | **34.4 (15.9; 74.5)** |
|  |  | 2024 | NA | **35.7 (15.4; 82.8)** |
|  |  | 2025 | NA | **37.1 (14.9; 92.2)** |
|  |  | 2026 | NA | **38.4 (14.3; 103.0)** |
|  |  | 2027 | NA | **39.9 (13.8; 115.2)** |
|  |  | 2028 | NA | **41.4 (13.3; 129.1)** |
|  |  | 2029 | NA | **42.9 (12.7; 144.8)** |
|  |  | 2030 | NA | **44.5 (12.2; 162.5)** |
|  | total | 2013 | 23.9 | **21.9 (13.5; 35.6)** |
|  |  | 2014 | 21.7 | **22.7 (14.4; 35.8)** |
|  |  | 2015 | 26.8 | **23.6 (15.3; 36.4)** |
|  |  | 2016 | 19.7 | **24.4 (15.9; 37.4)** |
|  |  | 2017 | 22.5 | **25.3 (16.4; 39.0)** |
|  |  | 2018 | 23.5 | **26.2 (16.6; 41.2)** |
|  |  | 2019 | 31.7 | **27.1 (16.7; 44.0)** |
|  |  | 2020 | NA | **28.1 (16.7; 47.5)** |
|  |  | 2021 | NA | **29.1 (16.5; 51.5)** |
|  |  | 2022 | NA | **30.2 (16.2; 56.3)** |
|  |  | 2023 | NA | **31.3 (15.8; 61.7)** |
|  |  | 2024 | NA | **32.4 (15.5; 67.9)** |
|  |  | 2025 | NA | **33.6 (15.0; 74.9)** |
|  |  | 2026 | NA | **34.8 (14.6; 82.8)** |
|  |  | 2027 | NA | **36.0 (14.1; 91.7)** |
|  |  | 2028 | NA | **37.3 (13.7; 101.7)** |
|  |  | 2029 | NA | **38.7 (13.2; 112.9)** |
|  |  | 2030 | NA | **40.1 (12.8; 125.4)** |
